# A Hotspot of TTX Contamination in the Adriatic Sea: Study on the Origin and Causative Factors

**DOI:** 10.3390/md21010008

**Published:** 2022-12-22

**Authors:** Simone Bacchiocchi, Debora Campacci, Melania Siracusa, Alessandra Dubbini, Stefano Accoroni, Tiziana Romagnoli, Alessandra Campanelli, Francesco Griffoni, Tamara Tavoloni, Stefania Gorbi, Cecilia Totti, Arianna Piersanti

**Affiliations:** 1 Istituto Zooprofilattico Sperimentale Umbria e Marche “Togo Rosati”, Via G. Salvemini, 1, 06126 Perugia, Italy; 2 Centro di Referenza Nazionale per il Controllo Chimico e Microbiologico dei Molluschi Bivalvi Vivi, Istituto Zooprofilattico Sperimentale Umbria e Marche “Togo Rosati”, Via Cupa di Posatora, 3, 60131 Ancona, Italy; 3 Dipartimento di Scienze della Vita e dell’Ambiente, Università Politecnica delle Marche, Via Brecce Bianche, 12, 60131 Ancona, Italy; 4 Consiglio Nazionale delle Ricerche, CNR-IRBIM, Largo Fiera della Pesca, 2, 60125 Ancona, Italy

**Keywords:** tetrodotoxin, mussels, shellfish, HILIC-MS, bivalves, emerging toxins, *Vibrio alginolyticus*, zooplankton, dinoflagellates

## Abstract

Tetrodotoxins (TTXs), the pufferfish venom traditionally associated with Indo-Pacific area, has been reported during last decades in ever wider range of marine organisms and ever more geographical areas, including shellfish in Europe. Wild mussels (*Mytilus galloprovincialis*) grown in the Marche Region (N Adriatic Sea, Italy) were shown to be prone to TTX contamination during the warm season, with a suspected role of *Vibrio alginolyticus* characterized by non-ribosomal peptide synthetase (NRPS) and polyketide synthase (PKS)-encoding genes. This work aimed to deepen the knowledge about the toxin’s origin and the way through which it accumulates in mussels. A two-year study (spring–summer 2020–2021) confirmed the recurrent presence of TTX (11–68 µg kg^−1^) in the official monitored natural mussel beds of the Conero Riviera. During 2021, a supplementary nonroutine monitoring of a natural mussel bed in the same area was carried out weekly from June until August for TTXs and/or the presence of *V. alginolyticus*. Biotic (mussels, mesozooplankton, worms and phytoplankton); abiotic (water and sediment) matrices and phytoplankton assemblage characterizations were studied. Mussels showed relevant TTX contamination levels (9–296 µg kg^−1^) with extremely rapid TTX accumulation/depletion rates. The toxin presence in phytoplankton and its distribution in the different mussel tissues supports its possible exogenous origin. The *V. alginolyticus* count trend overlaps that of TTX contamination in mussels, and similar trends were reported also for some phytoplankton species. The role of *V. alginolyticus* carrying NRPS or PKS genes as a possible TTX source and of phytoplankton as a “potential vector” should therefore be further investigated.

## 1. Introduction

Tetrodotoxin and its more than 30 analogs (TTXs) are the neurotoxins responsible of the severe and sometime lethal pufferfish poisoning in humans. They are alkaloids with a guanidinium moiety able to bind and block the voltage-gated sodium channels on muscular and neuronal cell membranes and thereby interfere with the nerve pulse transmission [1,2,3,4,5,6]. Symptoms ranging from mild, as numbness on mouth, to extremely severe, as paralysis or even death, occur rapidly after ingestion of contaminated seafood. Historically associated only to pufferfish, TTXs have been for a long time a health concern for the Indo-Pacific area (Japan, Thailand, Bangladesh and the Philippines), where the consumption of this type of fish causes dozens of deaths every year [7]. However, in the last decades, TTXs have been reported in ever more geographical areas [8,9,10,11,12] and a wider range of marine organisms belonging to fish, mollusks, crustaceans, echinoderms and worms [13,14,15,16,17,18,19]. The recent detection of TTXs in bivalves and gastropods from diverse geographical areas within European waters (Greece, the UK, the Netherlands, Spain, Portugal and Italy) [8,9,10,11,12,20,21,22] grew concern in the Europe and EU commission requested European Food Safety Authority (EFSA) to provide a full risk assessment. 

The EFSA scientific opinion [23], reporting the knowledge state of the art on the topic, contained substantially two main conclusions: (i) the need of more data on TTXs occurrence in mollusks in Europe for a better risk exposure assessment and (ii) the indication of a provisional concentration threshold set at 44 μg TTX equivalents kg^−1^ shellfish meat. Despite the TTXs’ chemical structures and the toxicity mechanism are already well known, this toxin group is still deeply investigated because its origin, biosynthetic pathway, and the mechanism of its accumulation in organisms are still uncertain. TTXs production by a variety of bacterial species of the genus *Vibrio, Bacillus, Pseudomonas, Actinomyces,* and *Micrococcus* has been widely documented [24,25,26,27], while only a few authors have suggested the possible involvement of microalgal dinoflagellates [10,13,28]. Attempts to describe the biosynthetic pathway of TTX in bacteria, even if not fully conclusive, hypothesized the involvement of enzymes such as non-ribosomal peptide synthetase (NRPS) and polyketide synthase (PKS) [4,29]. Therefore, NRPS and PKS genes have been targeted in the protocols for detection of potential TTX-producing bacteria [9]. Assuming the bacterial origin of TTXs, what remains to be explained is how the very low amount of toxins synthesized in vitro by bacteria could explain the extremely high levels reported in some organisms [30]. Two hypotheses have been proposed: an enhanced TTXs synthesis by TTX producer bacteria inside their endosymbiotic hosts (endogenous hypothesis) or an accumulation of toxins throughout the food web (exogenous hypothesis) [31]. 

Mussels (*Mytilus galloprovincialis*) growing on natural beds along the rocky coasts of the Marche Region (N Adriatic Sea, Italy) showed to be prone to TTXs contamination, even if at low concentrations [22]. The warm season and the shallow water seemed to be causative factors in agreement with previous findings in other geographical areas [9,11,32]. The possible correlation of TTX contamination with the presence in mussels of *Vibrio alginolyticus* containing NRPS and/or PKS genes was suggested, but more evidence is still needed [22]. This work aims to give further insights about the origin and the way TTXs accumulate in mussels. During spring-summer 2020, mussels from natural beds (harvested in the frame of the official mussel monitoring plans) were analyzed by HILIC LC-MS/MS to confirm the seasonal accumulation of TTXs. During spring-summer 2021, several environmental matrices and organisms were sampled from one supplementary sampling site (not included in the monitoring plan) “Molo Portonovo” along the Conero Riviera (Marche Region). Water, sediment, phytoplankton, mussels and other symbiotic organisms found among mussels specimens (mesozooplankton and worms) were weekly harvested and analyzed to investigate the TTXs origin, distribution in the ecosystem and mechanism of accumulation. Water, sediments, phytoplankton and mussels were also analyzed to assess the presence of *V. alginolyticus*, aiming to investigate its role in TTXs contamination in mussels. Contaminated mussel digestive glands, gills and mantles were dissected, pooled and analyzed separately in order to evaluate the toxins accumulation and distribution in the three tissues, also as clue of endogenous or exogenous origin of TTXs. Phytoplankton community was also analyzed to identify species acting as potential vectors of TTXs. The finding of this work represents a step forward in the knowledge of mechanisms and causative factors involved in TTXs mollusks contamination, fostering both future research and developing effective control plans.

## 2. Results

### 2.1. TTX in Mussels Natural Beds

**Figure 1:**
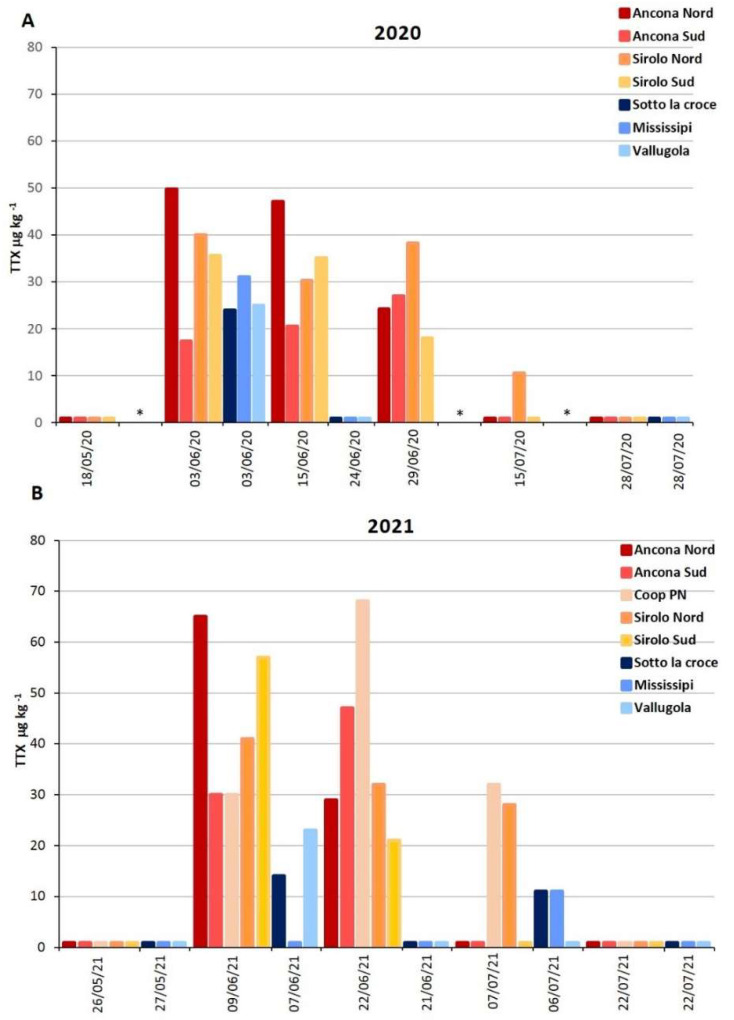
TTX levels (µg kg^−1^) in mussel samples from coastal area of the Marche Region during 2020 (**A**) and 2021 (**B**) (* sites not sampled).

During 2020, 74 mussel samples were harvested biweekly starting in May until October from their natural beds in the Marche Region (Table S1). In 16 samples (21.6%), TTX was higher than the limit of quantification LOQ of 8 µg kg^−^^1^ (11–50 µg kg^−^^1^, Figure 1), and 2 of them (2.7%), from Conero Riviera natural beds, showed levels above the EFSA safety threshold of 44 μg TTX equivalents kg^−^^1^ (47–50 µg kg^−^^1^, Table S1). The contaminated samples from Pesaro natural beds showed TTX levels (24–31 µg kg^−^^1^) comparable with those found in the Conero Riviera, but the contamination episode lasted for a shorter time span, resulting in only one sampling with quantified TTX levels (Table S1). The contaminated samples were collected between June and the first half of July and contained only the TTX itself. None of the other TTX analogs included in the method were detected.

During 2021, a total of 64 samples were harvested from May until September and 16 (25%) showed TTX levels > LOQ (11–65 µg kg^−^^1^), with 4 of them (6.3%) belonging to the Ancona area, at levels above the EFSA warning limit (47–64 µg kg^−^^1^, Table S1). The Pesaro contaminated samples showed lower levels (11–23 µg kg^−^^1^) (Table S1, Figure 1). Additionally, in 2021, all the contaminated samples were collected between June and early July (Figure 1), and only the parent toxin TTX was detected.

### 2.2. TTX Distribution in Mussel Tissues

Among all the samples harvested during 2021, the ten collected in the Ancona area on 9 and 22 June 2021 were selected because of their higher contamination levels and dissected to study the toxin’s compartmentalization in the different tissues.

Considering that a mussel specimen, as experimentally verified, is, on average, composed by weight by 15% digestive gland (DG), 15% gills (G), 30% Mantle (M) and 40% remaining tissues (RT), was calculated the distribution of total TTX in the tissues (compartmentalization). A homogeneous distribution of TTX among tissues would result in a toxin distribution (calculated as the concentration of the toxin in the specific tissue divided by the total toxin in mussel expressed in %) in the tissues compartment, equal to the mussel composition (15% of the toxin in DG, 15% in G and 30% in M and 40% in RT).

What came out from the study was that two different distribution profiles were encountered for samples of 9 and 22 June, none corresponding to the percentage of tissue distribution in mussels, meaning accumulation in certain specific tissues. 

**Figure 2:**
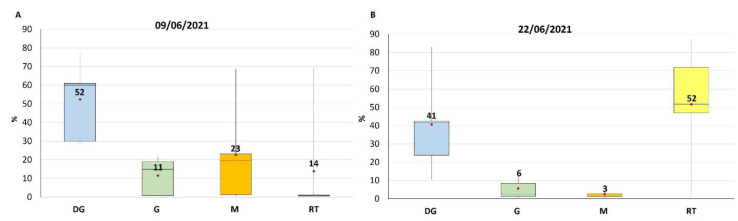
Box and whisker plot of TTX distribution in different mussel tissues expressed as percentage %, measured in samples collected on 9 June 2021 (**A**) and on 22 June 2021 (**B**). DG = Digestive Gland, G = Gills, M = Mantle, RT = Remaining Tissues.

The two distribution profiles are showed, as percentage (%), in Figure 2, the data were reported in Table S2. In both cases, albeit to a different extent, TTX showed a preferential accumulation in DG (52% for samples of 9 June and 41% for those of 22 June), but while in the samples of 9 June the remaining TTX was present consistently also in G and M (11 and 23% respectively), in those of 22 June the toxin was almost exclusively in the RT (52%).

### 2.3. TTX in Biota from Molo Portonovo

**Figure 3:**
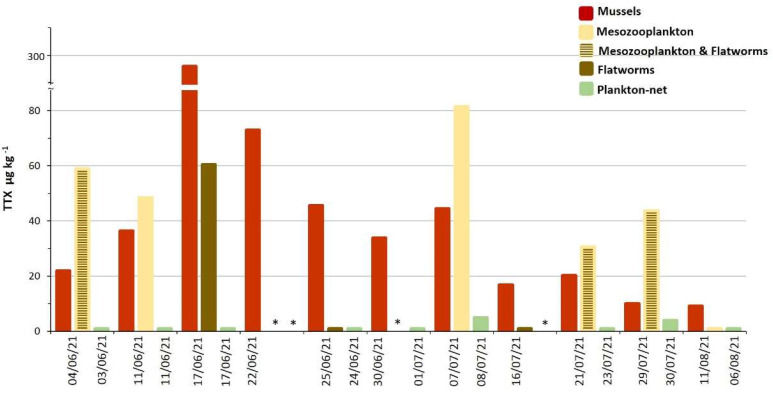
TTX contamination in various matrices from Molo Portonovo (MP) sampling point during 2021 (* sites not sampled).

Mussels from the sampling point Molo Portonovo (MP) showed a detectable amount of TTX from the first sampling day (4 June 2021), reaching a maximum of 296 µg kg^−1^ on June the 17th followed by a detoxification period lasted more than two months (Table S3, Figure 3). The TTX accumulation rate by mussels was slow during the first week (+4.7% of the maximum contamination) and extremely fast in the second one (+87.9%). After the maximum level was reached, the TTX depletion showed a fast trend during the first week (−75.4%), followed by a much slower toxin elimination rate (−21.6%) in the following five weeks (Table S3, Figure 3). The analysis of flatworms and mesozooplankton collected at MP mussel bed showed in some cases detectable amounts of TTX. Among flatworm samples only collected on 17 June, the period of maximum contamination in mussels, showed measurable levels of TTX (60 µg kg^−1^, Table S3, Figure 3). In some cases, the flatworms and mesozooplankton samples were not collected separately. When mesozooplankton was analyzed alone or with flatworms, the TTX was always measurable at levels higher than those found in mussels, except in the sampling of 11 August 2021 (Table S3, Figure 3). The analysis of a phytoplankton pellet collected with a net (plankton-net) through the water column barely showed detectable TTX levels (4–5 µg kg^−1^) in the samples collected on 8 and 30 July 2021.

### 2.4. Vibrio alginolyticus in Biotic and Abiotic Samples

During 2021, 34 mussel samples from the natural beds of the Conero Riviera were analyzed for *Vibrio* spp. In 32 samples (94%), at least one bacterial strain with *V. alginolyticus* characteristic colonies was isolated (Table S4). All isolates were then confirmed as *V. alginolyticus* by PCR. No other characteristic colonies belonging to *Vibrio* species were isolated. All the *V. alginolyticus* strains were analyzed for the NRPS and PKS genes and strains with at least one target gene was isolated from 15 mussel samples (44.1%). In 13 (38.2%) of these only the NRPS gene was identified, in 2 (5.9%) only PKS, while in no samples were both genes detected (Table S4).

**Figure 4:**
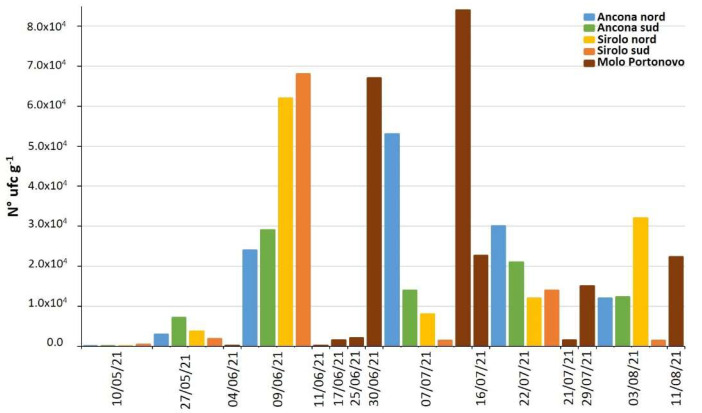
*V. alginolyticus* counts on mussel from natural beds of the Conero Riviera during 2021.

**Figure 5:**
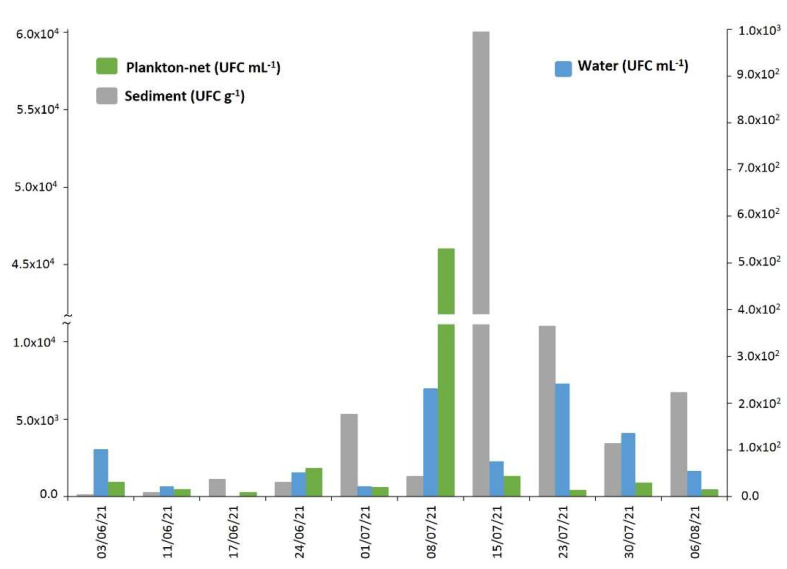
*V. alginolyticus* count on various matrices from MP natural bed during 2021.

As regards the *V. alginolyticus* count in mussels from the Conero Riviera natural beds, a significant increase, over three orders of magnitude (from 10^1^ to 10^4^ UFC g^−1^), was observed between the first sampling (on 10 May 2021) and that of 9 June 2021 (Table S4, Figure 4). Subsequently, until the last sampling on 11 August 2021, the contamination levels remained high, albeit fluctuating among the sampling points. The *Vibrio* analyses carried out in water, sediment and net-phytoplankton collected at the MP sampling site showed a similar trend with the maxima recorded during the month of July (Table S5, Figure 5).

### 2.5. Phytoplankton Characterization

**Figure 6:**
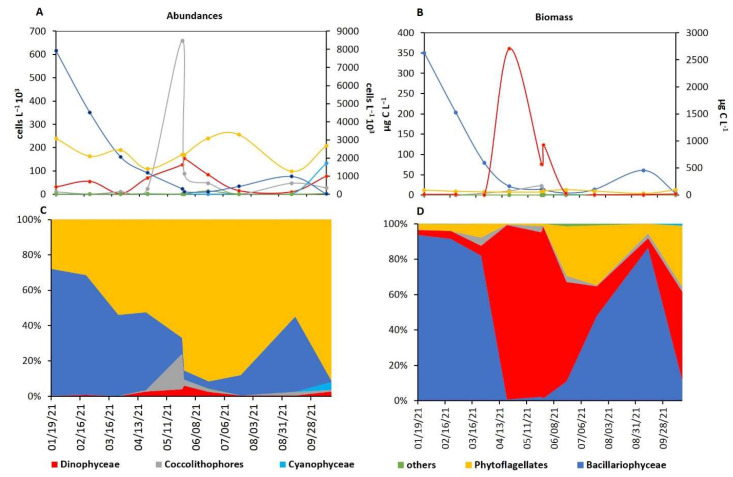
Trend in absolute abundances (**A**), biomass (**B**) and percent (**C**,**D**) of phytoplankton composition in PN in 2021: Bacillariophyceae, Coccolithophores, Cyanophyceae, others (left y-axis in **A**,**B**), phytoflagellates (right y-axis in **A** and left y-axis in **B**) and Dinophyceae (right y-axis in **A** and **B**).

Studying the phytoplankton annual trend in the Portonovo (PN) station, it is possible to observe that dinoflagellates (the phytoplankton group most suspect as being involved in TTX contamination of mussels) showed the highest abundances in the period from May to June (i.e., when TTX contamination in mussels was recorded, Figure 6). Indeed, dinoflagellate represented the major component of the phytoplankton in terms of biomass from April to July (Figure 6A), where the April peak of the biomass was due to the high abundances of *Noctiluca scintillans* (a big-sized heterotrophic dinoflagellate that markedly affects the biomass values [33].

Moreover, comparing the dinoflagellate abundance and biomass between the period when TTX mussel contamination occurred and the rest of the year, significantly higher values were recorded during the former (105.000 ± 30.541 cell L^−1^ and 120.837 ± 34.911 µg C l^−1^) than the latter (37.735 ± 32.930 cells L^−1^ and 36.720 ± 30.181 µg C L^−1^, Tukey’s HSD test both *p*-level < 0.05). No significant differences were observed for the other phytoplanktonic groups during the observation period. Among dinoflagellates, *Prorocentrum micans* and *Protoperidinium* cf. *steinii* showed significant higher abundance (868.0 ± 410.8 and 128.3 ± 46.6 cells L^−1^, respectively) and biomass (1.934 ± 0.915 and 0.409 ± 0.149 µg C L^−1^, respectively) values during TTX mussel contamination with respect to the rest of the year (124.0 ± 174.0 and 0.0 ± 0.0 cells L^−1^ Tukey’s HSD test *p*-level < 0.01 and 0.001, respectively for the abundances and 0.276 ± 0.388 and 0.0 ± 0.0 µg C L^−1^, Tukey’s HSD test *p*-level < 0.01 and 0.001, respectively, for the biomass). *Protoperidinium* cf. *steinii* (a heterotrophic dinoflagellate with a maximum abundance and biomass of 81 cells L^−1^ and 0.268 µg C L^−1^ on 17 June 2021) was detected in the water column only in the period of TTX contamination in mussels, while *Prorocentrum micans* bloomed with abundances of up to 3821 cells L^−1^ (corresponding to 11.749 µg C L^−1^) on 11 June 2021. *Prorocentrum cordatum* (suspected producer of TTX) [9,33,34] was recorded with a maximum abundance of 6726 cell L^−1^ (corresponding to 1.217 µg C L^−1^) in late May, just before the period when the mussels were found to be TTX-contaminated.

Studying more in detail the dinoflagellate (Dinophyceae) community during the TTX mussel contamination, the total abundances of the obligate heterotrophic species showed significant positive correlation with TTX amount in contaminated mussels (*r* = 0.860, *n* = 10, *p*-level < 0.01). Considering each taxon separately (Table S6), several taxa (not only among the obligate heterotrophs) showed significant positive correlation with the TTX amount in contaminated mussels: *Tripos extensum, Gonyaulax spinifera, Oxytoxum crassum, Protoperidinium* cf. *steinii, Protoperidinium pellucidum, Protoperidinium* sp. and *Noctiluca scintillans.* Moreover, all these dinoflagellates showed a significant positive correlation with the PO_4_^3−^ values.

In the MP station, during the study period, the water temperature showed an increasing trend, from 17.1 °C recorded on 3 June 2021 to 26.5 °C recorded on 30 July 2021. The salinity, on the other hand, was almost constant between 35.9 and 36.8, with the only exception recorded on 11 June 2021 when the salinity showed a sharp decrease to 34.3. During the period of maximum TTX accumulation in mussels, the water temperature ranged between 20 and 25 °C and salinity ranged from 34.3 to 36.

Regarding nutrients, the concentrations of Dissolved Inorganic Nitrogen (DIN) ranged from 1.07 to 5.00 µM and those of PO_4_^3−^ from 0.03 to 0.31 µM, with the maximums recorded on 17 June. During this period, the N: P ratio ranged from 12 to 109.

**Figure 7:**
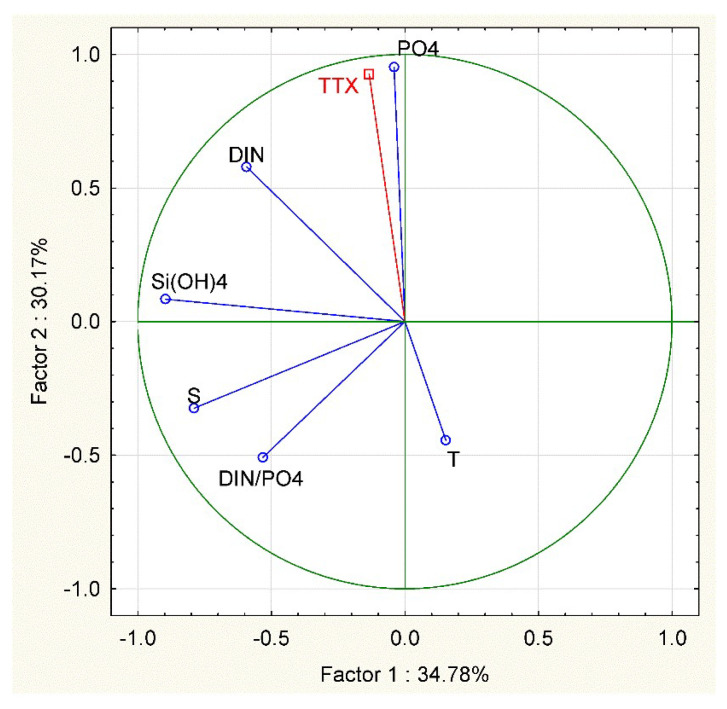
Principal Component Analysis (PCA) based on correlation matrix of the environmental variables and TTX concentration in mussels used as supplementary variables in MP in 2021: T = temperature (°C), S = salinity, DIN = Dissolved Inorganic Nitrogen (µM), PO_4_^3−^ = orthophosphate (µM), DIN/PO_4_^3−^ = dissolved inorganic N:P and TTX = tetrodotoxin (µg Kg^−1^).

Among the environmental parameters, TTX concentration in mussels showed a significant positive correlation with the PO_4_^3−^ values (r = 0.920, n = 10, *p*-level< 0.001, Figure 7).

## 3. Discussion

The two-year study on mussels harvested from natural beds, located along Marche coasts during Official Control, brought about the hypothesis that the TTX contamination is a recurrent phenomenon occurring between June and August, as previously reported [22]. Its incidence was greater in mussels of the Ancona area (Conero Riviera) rather than in those of the Pesaro’s, suggesting the Conero Riviera as a possible hotspot for TTX accumulation in shellfish. The TTX levels found in mussels were always moderate, with a percentage of the samples containing a measurable concentration of toxins ranging around 22–23%, only a few (<5%) levels above the EFSA threshold. Thus, TTX in commercialized wild mussels from the Marche Region does not represent an imminent concern for consumers, even if the TTX levels seem to increase.

This study focused on 2021 on mussels from “Molo Portonovo” (not included in Official Controls) that showed a higher toxin content (max 296 µg kg^−1^), among the highest ever found in Europe in bivalves, and a longer lasting contamination period. This sampling point was characterized by shallow waters (20–40 cm), and, during the period of maximum TTX accumulation in mussels, the water temperature ranged between 20 and 25 °C and salinity ranged from 34.3 to 36. These observations suggests that environmental factors such as strong solar radiation and relatively high water temperature may favor TTX accumulation in mussels, as already hypothesized in previous studies [9,32]. The Portonovo Bay is an area with relevant touristic activities during the summer, where wild mussels are easily accessible and heavily exploited by tourist fishers. The MP mussel natural bed, in particular, is very close to the beach and located in a swallow water area; therefore, the considerable amount of TTX detected in June (about seven-fold the EFSA safe threshold) may represent a cause for concern.

The TTX accumulation/depletion rates in mussels were extremely rapid, with a one-week scale for 75–80% of the maximum TTX concentration accumulation/detoxification. Similar trends have already been reported by previous studies on bivalves from the United Kingdom [32] and France [35].

The TTX distribution in mussel-contaminated tissues showed, in the early stage, a preferential accumulation in DG followed by G and M. After about two weeks, DG remains the preferential accumulation tissue, but the levels in G and M were significantly lower, probably as a consequence of a redistribution in the whole organism and/or different metabolization rates.

The described toxin distribution in mussels and its changes over exposure time seems to confirm the hypothesis of an exogenous origin of TTX: the toxin is produced externally, reaches the mussels through the diet, accumulates initially in those tissues involved in the filtration (G, M) or digestion (DG) processes and, only after, it redistributes in the rest of tissues. The hypothesis is supported by the detectable levels of TTX in mesozooplankton and in net-plankton samples collected at the same time mussel samples were harvested.

Moreover, the relevant zooplankton contamination levels, taking into account the little amount of material analyzed, suggests that these organisms likely play an important role in the toxin trophic transfer acting as reservoir and vector to mussels. Only in one case a sample of flatworms showed detectable levels of TTX when mussels reached the maximum contamination. Some authors in the literature have reported how pufferfish and other fishes acquire TTX by feeding on toxic planocerid flatworms [36,37,38] and that bivalves can also be contaminated by feeding on planocerid larvae [39]. Toxic flatworms seem to be restricted to the specific *Planocera* lineages [40]. Flatworms isolated in the MP natural bed were identified as *Stylochus mediterraneus,* a proven mussel *Mytilus galloprovincialis* predator [41]; then, it can be hypothesized they accumulate TTX by eating contaminated mussels. 

The analysis of phytoplankton seems to suggest a potential role of some species in TTX contamination of mussels. Analyzing the phytoplankton annual trend, it is possible to observe that dinoflagellates showed the highest abundances exactly in the period in which TTX contamination was recorded in mussels. Considering that many dinoflagellates are heterotrophic, and the majority are mixotrophic (characteristics that allow them to feed on potentially TTX-producing bacteria), their possible role as vectors in the TTX contamination of mussels might be hypothesized. Indeed, the total abundances of obligate heterotrophic dinoflagellates resulted in being positively correlated with the TTX levels in contaminated mussels. In particular, among those dinoflagellates, i.e., *Protoperidinium* cf. *steinii, Protoperidinium pellucidum, Protoperidinium* sp. and *Noctiluca scintillans,* the former was detected only in this period. Moreover, even some non-obligate heterotrophic dinoflagellates could play a possible role in TTX mussels contamination: (i) *Tripos extensum*, *Gonyaulax spinifera* and *Oxytoxum crassum* resulted in being positively correlated with TTX in contaminated mussels, (ii) *Prorocentrum cordatum* was sometimes associated with TTXs accumulation in mussels [7,10,34], and in this study, its abundance in water reached its maximum just before the period in which mussels were found to be contaminated by TTX and (iii) *Prorocentrum micans* bloomed in concomitance with the period of mussel contamination. Overall, these observations prompt further investigations in the coming years, because it is not still clear if certain species are directly involved in this process or if it is just a biomass issue, i.e., if every mixotrophic or heterotrophic dinoflagellate can act as vector if it reaches a certain value of biomass when TTX-producing bacteria are present in the environment. Even the positive correlation found between TTX in mussels and PO_4_^3−^ levels in the summer, when P is the limiting factor for phytoplankton reproduction [42,43], highlights that those environmental parameters favoring the phytoplankton (in this case, the increase of PO_4_^3−^) can indirectly influence the TTX contamination in mussels.

During both years of the study, *V. alginolyticus* was found in almost all the mussels samples from the natural bed (94.1%). *V. alginolyticus* was also isolated from the other biotic and abiotic matrices collected in the same habitat. This result was certainly expected, as it is known that *Vibrio* are the most abundant bacteria in marine environments [44] and that *V. alginolyticus* is the predominant species along the Adriatic coast, followed by *V. parahaemolyticus*, *V. cholerae* and *V. vulnificus* [45]. Furthermore, *V. alginolyticus* strains isolated from roughly the half of the samples (44.1%) contained NRPS or PKS genes. 

In order to investigate a possible correlation between *V. alginolyticus*, a TTX potential producer and measurable toxin levels in the mussels, the microbiological and biomolecular results were compared with the chemical ones.

Firstly, the accumulation of TTX and the contamination by *V. alginolyticus* in mussels and in the entire natural beds’ ecosystem followed fairly similar trends, reaching the maximum levels between June and July. This is surely not sufficient to demonstrate a correlation between the two phenomena, but it highlights that they are certainly favored by the same environmental factors (temperature, water depth and solar radiation). Thus, the origin of the toxin can reasonably be traced back to microorganisms (bacteria), with ecophysiological characteristics similar to those of *V. alginolyticus*.

Furthermore, out of the 27 samples submitted to both chemical and microbiological analyses, 9 (33%) were found to be simultaneously contaminated by *V. alginolyticus* and by TTX, which is 60% of the samples [17] contaminated by *V. alginolyticus* carrying the NRPS/PKS genes and 75% of the samples contaminated by TTX [14]. This is not itself conclusive of a correlation between the two phenomena, but it certainly underlines the need for further investigation.

Another relevant take home result of this study is that an effective monitoring plan able to promptly report TTX contamination trends in mussels belonging to a given area should focus on those sites with environmental features known to lead to TTX accumulation (shallow water) during the warm season and with a higher sampling frequency (one week or less).

## 4. Materials and Methods

### 4.1. Sampling

**Figure 8:**
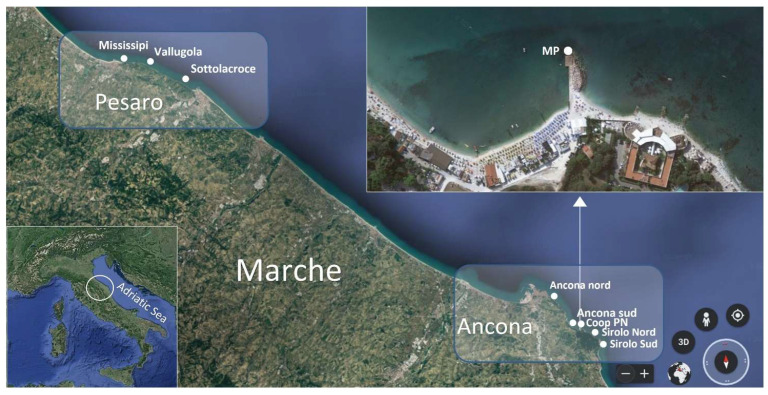
Sampling sites along the Marche coast. Seven officially monitored natural beds: Mississippi, Vallugola, Sotto lacroce (Pesaro area), Ancona nord, Ancona sud, Sirolo nord, Sirolo sud, the breeding site Coop PN and the not routinely monitored natural bed Molo Portonovo (MP) (Ancona area). Images (data SIO, NOAA, U.S. Navy, NGA, GEBCO; image Landsat/Copernicus) are from Google Earth.

A total of 138 mussel samples (*Mytilus galloprovincialis*) were collected fortnightly from May to October during 2020 (74 samples) and from May to September during 2021 (64 samples) from seven natural beds along Marche region and a mussel breeding site (only during 2021) included in the biotoxin regional monitoring plan (N Adriatic Sea, Italy). In natural beds, mussels live clinging to rocks generally in shallow water (<5 m) near the cliffy coast. Three natural beds are located in the Pesaro area (Sottolacroce, Vallugola and Mississippi) and four in the Ancona’s along the Conero Riviera (Ancona Nord, Ancona Sud, Sirolo Nord and Sirolo Sud) (Figure 8). In the mussel farm (Coop PN), located in the Portonovo Bay (Ancona), mussels grow attached to a submerged concrete structure in shallow water (<5 m). Each mussel sample consisted of about 20–30 commercially sized mussels (5–7 cm). Samples were used both for chemical and microbiological analyses.

During 2021, from June to August, with a weekly frequency, sampling was carried out also in a further sampling station named “Molo Portonovo” (MP, 43°33′55″ N, 3°35′26″ E) (Figure 8). In each sampling day, the meteorological parameters were also recorded. A CTD Model 30 Handheld Salinity, Conductivity and Temperature System, YSI (Yellow Spring, OH, USA) was used to measure the surface temperature and salinity. Water samples were collected in polyethylene bottles for a nutrient analysis (50 mL), immediately filtered through GF/F Whatman filters (25 mm) and stored in triplicate in 4 mL polyethylene bottles at −22 °C until the analysis. In MP collected mussels, water, net-phytoplankton and sediment samples sent to TTXs and the *Vibrio* analysis. Mussels (11 samples) were taken from the natural bed. Surface sea water was collected with sterile 50 mL Falcon. Sediment samples were collected using sterile Falcon tubes directly on the sediment surface by SCUBA divers and stored at 4 °C until analysis. Phytoplankton was sampled through a net (mesh diameter 20 µm). 

Phytoplankton samples for species identification and counting were collected at surface with a 250 mL dark glass bottle and preserved with 0.8% formaldehyde (prefiltered and neutralized with hexamethylenetetramine) [46]. Moreover, to appreciate the phytoplankton annual cycle in the study area, we used the 2021 data obtained from the monthly monitoring activity of Dipartimento di Scienze della Vita e dell’Ambiente (DISVA—Università Politecnica delle Marche) in a close area (station PN located at approx. 2 nM from the coast, 43°36.201′ N, 13°36.705′ E). 

### 4.2. Sample Treatment

Seawater (50 mL), sediment (50 g) and phytoplankton-net samples were harvested; transported to the laboratory in refrigerated conditions (4 °C) and processed immediately for microbiological analysis. Phytoplankton-net samples were then centrifuged at 3.000× *g* for 10 min and pellets stored at −20 °C until chemical analysis. From mussels arrived in the laboratory, some organisms colonizing the mussel bed were harvested directly from the valves or from the water collected with mussels picking them up with tweezers or with a pipette. Flatworms, identified as *Stylochus mediterraneus* (1–3 specimens), and/or small crustaceans (5–10 specimens of 0.1–1.0 cm size, mesozooplankton) were sampled and stored at −20 °C until chemical analysis.

### 4.3. Phytoplankton Identification and Counting 

Identification and counting of phytoplankton were carried out using an inverted microscope (ZEISS Axiovert 135, Oberkochen, Germany) equipped with phase contrast, following the Utermöhl’s method [47]. Counting was carried out at 400× magnification, along transects or in random visual fields, depending on cell abundance and enabling the counting of a minimum of 200 cells. Moreover, half of the Utermöhl’s chamber was analyzed at 200× magnification for a more precise estimation of less abundant phytoplanktonic taxa. Phytoplankton taxa were grouped into major groups (diatoms, dinoflagellates, coccolithophores, phytoflagellates and cyanobacteria), and abundances were expressed as cellsL^−1^. Dinoflagellates were considered as a taxonomical group and both autotrophic and heterotrophic species were included in counting. Phytoflagellates are an informal group that includes haptophytes (except coccolithophores), cryptophytes, chrysophytes, dictyochophytes, raphidophytes, chlorophytes and euglenophytes. Phytoplankton biomass was estimated through cell biovolumes: single cells were measured using a micrometric ocular approximating cell shapes to geometrical figures [48]. Then, the carbon content for each taxon was derived by the mean biovolume, following [49], and the biomass was expressed as μg C L^−1^.

### 4.4. Chemical Analysis

#### 4.4.1. Chemicals and Standards

All the reagents were of analytical grade. Acetonitrile (LC-MS grade) and methanol (HPLC grade) were purchased from CARLO ERBA Reagents S.r.l. (Cornaredo, Milan, Italy). Ammonium hydroxide (≥25% in water, LC-MS grade) was purchased from Merck (Darmstadt, Germany), formic acid (LC-MS grade) from VWR International (Radnor, PA, USA) and glacial acetic acid (reagent grade) from Sigma-Aldrich (Steinheim, Germany). Ultrapure water was produced by the Milli-Q water purification system (Millipore Ltd., Bedford, MA, USA).

Tetrodotoxin Certified Reference Material (CRM-003-TTXs) was obtained from Cifga Laboratory (Lugo, Spain). The CRM was a mixture of tetrodotoxin (25.9 ± 1.2 µg g^−1^) and 4,9-anhydro tetrodotoxin (2.99 ± 0.16 µg g^−1^).

A stock solution in water was prepared from the CRM. Matrix-matched calibration standards were obtained for dilution of the stock solution using and extract of a blank sample. Moreover, the stock solution was used to prepare quality control. 

#### 4.4.2. TTXs Extraction 

##### Mussels, Flatworms and Mesozooplankton

The TTXs extraction from mussels, flatworms and mesozooplankton was performed following the EU-SOP “Determination of Tetrodotoxin by HILIC-MS/MS” [50], with some modifications. The clean-up step was removed, and only the final dilution was applied to reduce matrix effect. Moreover, the applicability of the method was extended to other matrices such as flatworms and mesozooplankton. The details are described below.

Bivalves were opened immediately once they arrived in the laboratory. Sand and solid residues were removed under running water, and the mussels were taken out of the shells and drained on a net. For each sample, about 150 g of whole flesh, was pooled and finely homogenized by an Osterizer blender. Ten specimens of the ten contaminated mussel samples harvested on 9 and 22 June from the Conero Riviera natural beds were dissected, and the DG, G and M were pooled for each sample and weighted to calculate their contribution to the whole body. The pools were then finely homogenized by Ultra Turrax (T25 basic IKA_WERKE) and analyzed separately in order to investigate the tissues distribution of TTXs. 

Samples containing flatworms and/or mesozooplankton were finely homogenized by Ultra Turrax. Homogenates (5.0 ± 0.1 g for mussels or mussel tissues (2.0 ± 0.1 g for flatworms and 0.5 ± 0.01 g for mesozooplankton) were extracted with 5 mL (or 2 mL for flatworms and mesozooplankton) of acetic acid (1% *v*/*v*), vortex-mixed for 3 min and placed in a boiling water bath (100 °C) for 5 min. The extract was cooled to room temperature, vortex-mixed for 3 min and centrifuged at 3000× *g* for 10 min. Supernatant (1 mL) was transferred to a microcentrifuge tube, to which 5 μL ammonium hydroxide was added, and the sample was vortex-mixed for 3 min and centrifuged at 10,000× *g* per 1 min. The final extract was diluted (1:2) with a solution of acetonitrile (80% *v*/*v*) containing acetic acid (0.25% *v*/*v*), filtered through a 0.22 μm syringe filter and analyzed by HILIC-MS/MS.

##### Net-Phytoplankton

Phytoplankton-net pellets (0.1 ± 0.01 g) were extracted with 2 mL of acetic acid (1% *v*/*v*), vortex-mixed for 3 min and bath-sonicated for 10 min. After sonication, the aliquots were centrifuged for 10 min at 2.500× *g* (4 °C), and the supernatants were transferred to a 100 mL evaporation flask. Pellets extraction was repeated three times, and the supernatants were combined and evaporated to dryness by a rotavapor (R-114 Büchi, Flawil, Switzerland). The residue was reconstituted in 1 mL of acetonitrile (80% *v*/*v*)-containing acetic acid (0.25% *v*/*v*) and filtered through a 0.22 μm syringe filter (Minisart, Sartorius, Germany) for a HILIC-MS/MS analysis.

#### 4.4.3. HILIC-MS/MS Analysis

The chromatographic separation was achieved according to the EU-SOP “Determination of Tetrodotoxin by HILIC-MS/MS” [50]; the details are given in Table S7.

Instrumental analysis was performed on the ACQUITY I-Class-Xevo TQ-S micro IVD system (Waters, Milford, MA, USA) equipped with electrospray ionization (ESI) source and operated in positive mode. Infusion experiments were performed on TTX CRM to optimize the mass parameters. Nine analogs (Table S7) were monitored via Multiple Reaction Monitoring (MRM), with two transitions selected for each toxin to allow correct identification and quantification. The MS acquisition method is described in Table S7. The unequivocal identification of the TTX chromatographic peak was obtained by retention time comparison and ion ratio verification for the two characteristic transitions in the samples and in a matrix-matched standard. All the other analytes, for which no reference materials were available, were identified by selecting specific transitions from the literature. All analogs were quantified with TTX, assuming an equimolar response. 

The LOQ and LOD adopted were, respectively, 8 µg kg^−1^ and 3 µg kg^−1^. The LOQ was estimated as the concentration giving a S/N ratio of 10 (for the least intense transition monitored) starting from the lowest level of the calibration curve. Afterwards, the estimated LOQ was experimentally confirmed by spiking blank mussel samples with the TTX CRM. The LOD used was calculated dividing the experimental LOQ by 3.3.

#### 4.4.4. Quality Control

Internal quality controls were included in a sample analysis: a blank mussel sample was spiked with TTX at the LOQ in each analytical batch. Accuracy in terms of recovery (R%) was calculated to check validation performances. In suspicious cases or in matrices different from bivalves, such as flatworms, mesozooplankton and net-phytoplankton, the samples were subjected to co-chromatography, in which they were spiked with a comparable amount of TTX to indubitably confirm the analyte identification.

### 4.5. Microbiological Analysis

#### 4.5.1. Vibrio Alginolyticus Isolation and Enumeration

Mussel samples were transported to the laboratory in refrigerated conditions and processed within 6 h from the collection. Samples were externally cleaned with potable water. In aseptic working conditions, about 10 individuals were opened and the flesh meat and intervalvular fluids were pooled together, and 25 g of bivalve sample were homogenized in a blender and tenfold diluted with alkaline saline peptone water (ASPW) (10^−1^ dilution). Sediment samples (10 g) were tenfold diluted with ASPW and vortex-mixed for 15 min at 2000× *g* with the Multi Reax (Heidolph, Germany) (10^−1^ dilution). Water and phytoplankton-net samples were vortex-mixed for 15 min at 2000× *g* with the Multi Reax. From seawater and net-phytoplankton raw samples and the 10^−1^ dilution of mussel and sediment samples further serial dilutions (10^−1^ to 10^−4^) were prepared in ASPW (1 + 9 mL); then, 1 mL of each dilution was surface-spread plated on TCBS agar (Difco Laboratories, Detroit, MI, USA) and incubated at 37 ± 1 °C for 18 ± 3 h. After incubation, yellow colonies growing on TCBS agar plates were counted (only plates with 3 to 150 colonies were considered) for *V. alginolyticus* enumeration [51,52]. The results were reported as UFC per gram of mussels/sediment or UFC per milliliter of water/phytoplankton-net sample.

#### 4.5.2. Vibrio Alginolyticus Molecular Identification and Characterization 

After incubation, colonies on TCBS agar were selected on the basis of distinctive morphology and color. At least five yellow colonies appearing on plates of the highest dilution were selected and subcultured on trypticase soy agar with 3% NaCl (TSAs, Oxoid) at 37 °C for 18 ± 3 h and submitted to a molecular assay. 

##### DNA Extraction—Operative Method

Bacterial colonies were suspended in 500 µL sterile distilled water, heated to 99 °C for 10 min and centrifuged at 13.200× *g* for 1 min [53]. The supernatant was either tested by PCR immediately or stored at −20 °C. Possible V. alginolyticus colonies were submitted to PCR analysis for the species-specific gyrB gene [54].

##### PCR Analysis

All the PCR amplifications were performed with a Mastercycler pro Thermal Cycler (Eppendorf).

- gyrB species-specific gene

Yellow colonies suspected of belonging to *Vibrio alginolyticus* strains were submitted to PCR analysis for detection of the species-specific gyrB [54] gene. PCR amplification protocol is described in Table S8. AlgF1 and AlgR1 primers (Invitrogen, Thermo Fisher, Waltham, USA.) were employed for the amplification of the gyrB gene fragment (568 bp) [54]. *V. alginolyticus* ATCC 33787 strain (American Type Culture Collection, Manassas, VA, USA) was used as positive control of amplification (CTRL^+^), while ultrapure distilled nuclease-free water was used as a negative amplification control (CTRL^−^) in all analytical batches. After the amplification reaction, the products were displayed by electrophoresis run in 1.5% agarose gel under UV light. The bacterial isolates were identified as *V. alginolyticus* when PCR amplification generated products of the expected size (568 bp) by comparison to a 100 bp DNA ladder molecular weight marker and the positive control strain.

- NRPS and PKS biosynthesis genes

The bacterial isolates identified as *V. alginolyticus* were subjected to PCR analysis for the presence of PKS and NRPS genes. PCR amplification protocols are described in Table S8 A2gamF and A3gamR primers were employed for the amplification of the NRPS gene fragment (300 bp) [55], and DKF and DKR degenerate primers were used for the amplification of PKS gene fragment (300 bp) [9,56]. *V. parahaemolyticus* ATCC 17802 strain was used as a positive control of amplification (CTRL^+^) for both target genes, while ultrapure distilled nuclease-free water was used as a negative amplification control (CTRL^−^) in all analytical batches. The *V. alginolyticus* and *V. parahaemolyticus* isolates were considered to be NRPS and/or PKS positive when PCR amplification generated products of the expected size (300 bp for both genes) by comparison to the molecular weight marker and the positive control strain after visualization by electrophoresis in 1.5% agarose gel under UV light.

### 4.6. Nutrient Analysis

The colorimetric method of Strickland and Parsons (1972) [57] was used to perform the analysis of nitrates (NO_3_^−^), nitrites (NO_2_^−^), ammonium (NH_4_^+^), phosphates (PO_4_^3−^) and silicates Si(OH)_4_ using an Autoanalyzer quAAtro Axflow. Detectability limits were 0.02 µM for NO_2_^−^, NO_3_^−^, NH_4_^+^ and Si(OH)_4_ and 0.03 µM for PO_4_^3−^. Dissolved Inorganic Nitrogen (DIN) concentration is intended as the sum of NO_2_^−^, NO_3_^−^ and NH_4_^+^ concentrations.

### 4.7. Statistical Analyses

Differences in abundance and biomass values of phytoplankton groups and dinoflagellate taxa between the period of TTX mussel contamination in 2021 and the rest of the year at PN and MP stations were assessed through a one-way analysis of variance (ANOVA). The abundances of dinoflagellate taxa were also tested for significant correlations (Pearson) with all recorded environmental parameters at MP station. A Tukey’s pairwise comparison test was also performed when significant differences for the main effect were observed (*p* < 0.05). 

A Principal Component Analysis (PCA) was performed on a correlation matrix of physical and chemical variables collected in MP station. Temperature, salinity, nutrient concentration and their ratios (DIN:PO_4_^3−^) were chosen to characterize the environmental conditions of the station and values of TTX concentration in mussels were used as supplementary variables. The statistical analyses were conducted using Statistica 12 software, (StartSoft Inc.,Tulsa, OK, USA).

The laboratory is accredited according to ISO/IEC 17025.

## 5. Conclusions

EFSA, in the scientific opinion on TTXs, recommends studies on the possible sources and critical factors that may lead to TTXs accumulation in marine bivalve mollusks and gastropods. Findings of this work confirmed that the Conero Riviera, in the coastal Ancona area, rich with wild mussels (*Mytilus galloprovincialis*), is a TTX hot spot in the northern Adriatic Sea. This is certainly a reason of concern for mussels’ consumers but also provides the opportunity to address the EFSA requests. The present study confirms that warm water, low water depth and strong solar radiation are potential critical factors for TTX accumulation and that TTX accumulation/depletion rates by mussels are extremely rapid processes. The presence of the toxin also outside the mussels and its distribution in mussel tissues strengthen the hypothesis of exogenous origin of TTX. Some results here reported seem to support the hypothesis that phytoplankton may have a key role in the TTX trophic transfer to mussels. The similar temporal trends of *V. alginolyticus*, often containing the PKS or NRPS genes, and TTX accumulation in mussels and other organisms encourages to pursue the attempt to trace back the source of TTX to this microorganism or microorganisms with similar characteristics.

## Data Availability

Not applicable.

## Supplementary Materials

The following supporting information can be downloaded at: https://www.mdpi.com/article/10.3390/md21010008/s1, Table S1: Tetrodotoxin (TTX µg Kg^−1^) in mussel samples from coastal area of the Marche region during 2020–2021; Table S2: Tetrodotoxin (TTX) distribution (%) in contaminated mussel tissues; Table S3: Tetrodotoxin (TTX µg Kg^−1^) contamination in various matrices from Molo Portonovo (MP) during 2021; Table S4: *Vibrio alginolyticus* count on mussel samples of the Conero Riviera during 2021; Table S5: *Vibrio alginolyticus* count on various matrices from “Molo Portonovo” sampling point during 2021; Table S6: Pearson’s correlation between dinoflagellate taxa abundances and TTX concentration in mussels, water temperature (T), salinity (S), DIN, Si(OH)_4_ and PO4^3−^ values. Values indicated in red are significant at *p* < 0.05, those in italic are significant at *p* < 0.01, those in bold italic are significant at *p* < 0.001. * indicates obligate heterotrophic dinoflagellates; Table S7: HILIC-MS/MS method for TTXs analysis: LC, MS parameters and transitions in Multiple Reaction Monitoring (MRM); Table S8: Reagents, reaction mix and protocols for PCR on Vibrio spp.
