# Supplementary material for: A Hotspot of TTX Contamination in the Adriatic Sea: Study on the Origin and Causative Factors
Source: Mar Drugs. 2022 Dec 22;21(1):8. doi: 10.3390/md21010008 (PMC9866420; doi:10.3390/md21010008)
Supplement: Supplementary file 1 [file marinedrugs-21-00008-s001.zip › Table S1.pdf]

Table S1. Tetrodotoxin (TTX μg Kg^{-1}) in mussel samples from coastal area of the Marche region during 2020-2021.

**2020-2021**									
PESARO				*CONERO RIVIERA*					
**Date**	*Sotto la Croce*	*Mississipi*	*Vallugola*	**Date**	*Ancona Nord*	*Ancona sud*	*Coop PN*	*Sirolo Nord*	*Sirolo Sud*
03/06/2020	**24**	**31**	**25**	06/05/2020	ND (< LOD)	ND (< LOD)	*	ND (< LOD)	ND (< LOD)
24/06/2020	ND (< LOD)	ND (< LOD)	ND (< LOD)	18/05/2020	ND (< LOD)	ND (< LOD)	*	ND (< LOD)	ND (< LOD)
28/07/2020	ND (< LOD)	ND (< LOD)	ND (< LOD)	03/06/2020	**50**	**17**	*	**40**	**36**
03/08/2020	ND (< LOD)	ND (< LOD)	ND (< LOD)	15/06/2020	**47**	**21**	*	**30**	**35**
18/08/2020	ND (< LOD)	ND (< LOD)	ND (< LOD)	29/06/2020	**24**	**27**	*	**38**	**18**
26/08/2020	ND (< LOD)	ND (< LOD)	ND (< LOD)	15/07/2020	ND (< LOD)	ND (< LOD)	*	**11**	ND (< LOD)
31/08/2020	ND (< LOD)	ND (< LOD)	ND (< LOD)	28/07/2020	ND (< LOD)	ND (< LOD)	*	ND (< LOD)	ND (< LOD)
21/09/2020	ND (< LOD)	ND (< LOD)	*	10/08/2020	ND (< LOD)	ND (< LOD)	*	ND (< LOD)	ND (< LOD)
30/09/2020	ND (< LOD)	ND (< LOD)	ND (< LOD)	27/08/2020	ND (< LOD)	ND (< LOD)	*	ND (< LOD)	ND (< LOD)
10/05/2021	ND (< LOD)	ND (< LOD)	ND (< LOD)	22/09/2020	ND (< LOD)	ND (< LOD)	*	ND (< LOD)	ND (< LOD)
26/05/2021	ND (< LOD)	ND (< LOD)	ND (< LOD)	08/10/2020	ND (< LOD)	ND (< LOD)	*	ND (< LOD)	ND (< LOD)
07/06/2021	**14**	ND (< LOD)	**23**	20/10/2020	ND (< LOD)	ND (< LOD)	*	ND (< LOD)	ND (< LOD)
21/06/2021	ND (< LOD)	ND (< LOD)	ND (< LOD)	10/05/2021	ND (< LOD)	ND (< LOD)	ND (< LOD)	ND (< LOD)	ND (< LOD)
06/07/2021	**11**	**11**	ND (< LOD)	27/05/2021	ND (< LOD)	ND (< LOD)	ND (< LOD)	ND (< LOD)	ND (< LOD)
22/07/2021	ND (< LOD)	ND (< LOD)	ND (< LOD)	09/06/2021	**65**	**30**	**30**	**41**	**57**
02/08/2021	ND (< LOD)	ND (< LOD)	ND (< LOD)	22/06/2021	**29**	**47**	**68**	**32**	**21**
16/08/2021	ND (< LOD)	ND (< LOD)	ND (< LOD)	07/07/2021	ND (< LOD)	ND (< LOD)	ND (< LOD)	ND (< LOD)	ND (< LOD)
				22/07/2021	ND (< LOD)	ND (< LOD)	**32**	**28**	ND (< LOD)
				03/08/2021	ND (< LOD)	ND (< LOD)	ND (< LOD)	ND (< LOD)	ND (< LOD)
				01/09/2021	ND (< LOD)	ND (< LOD)	ND (< LOD)	ND (< LOD)	ND (< LOD)

* sample not analysed
ND = not detected
LOD = Limit of detection (3.0 μg Kg^{-1})
LOQ = Limit of quantification (8.0 μg Kg^{-1})
