# Supplementary material for: A Hotspot of TTX Contamination in the Adriatic Sea: Study on the Origin and Causative Factors
Source: Mar Drugs. 2022 Dec 22;21(1):8. doi: 10.3390/md21010008 (PMC9866420; doi:10.3390/md21010008)
Supplement: Supplementary file 1 [file marinedrugs-21-00008-s001.zip › Table S2.pdf]

Table S2. Tetrodotoxin (TTX) distribution (%) in contaminated mussel tissues.

Site	Date	TISSUE DISTRIBUTION (%)			
		DG	G	M	RT
AN Nord	09/06/2021	**29**	**1**	**1**	**69**
AN Sud		**61**	**15**	**23**	**1**
Coop PN		**30**	**1**	**69**	**1**
Sirolo Nord		**60**	**19**	**20**	**1**
Sirolo Sud		**77**	**22**	**1**	**1**
AN Nord	22/06/2021	**83**	**15**	**1**	**1**
AN Sud		**43**	**9**	**2**	**47**
Coop PN		**11**	**1**	**1**	**87**
Sirolo Nord		**24**	**1**	**3**	**72**
Sirolo Sud		**42**	**2**	**4**	**52**

DG = Digestive Gland
G= Gills
M = Mantle
RT= Remaining tissues
