# Supplementary material for: A Hotspot of TTX Contamination in the Adriatic Sea: Study on the Origin and Causative Factors
Source: Mar Drugs. 2022 Dec 22;21(1):8. doi: 10.3390/md21010008 (PMC9866420; doi:10.3390/md21010008)
Supplement: Supplementary file 1 [file marinedrugs-21-00008-s001.zip › Table S3.pdf]

Table S3. Tetrodotoxin (TTX μg Kg^{-1}) contamination in various matrices from Molo Portonovo (MP) during 2021.

**Molo Portonovo (MP)** *2021*						
**Date**	***mussels***	***mesozooplankton***	***flatworms***	***mesozooplankton and flatworms***	**Date**	***Plankton-net***
04/06/2021	**22**	-	-	**59**	03/06/2021	ND (< LOD)
11/06/2021	**36**	**48**	*	*	11/06/2021	ND (< LOD)
17/06/2021	**296**	*	**60**	*	17/06/2021	ND (< LOD)
22/06/2021	**73**	*	*	*	24/06/2021	ND (< LOD)
25/06/2021	**46**	*	ND (< LOD)	*	01/07/2021	ND (< LOD)
30/06/2021	**34**	*	*	*	08/07/2021	**5**
07/07/2021	**45**	**81**	*	*	23/07/2021	ND (< LOD)
16/07/2021	**17**	*	ND (< LOD)	*	30/07/2021	**4**
21/07/2021	**20**	-	-	**31**	06/08/2021	ND (< LOD)
29/07/2021	**10**	-	-	**44**		
11/08/2021	**9**	ND (< LOD)	*	*		

- pooled sample
* sample not found
ND = not detected
LOD = Limit of detection (3.0 μg Kg^{-1})
