# Supplementary material for: A Hotspot of TTX Contamination in the Adriatic Sea: Study on the Origin and Causative Factors
Source: Mar Drugs. 2022 Dec 22;21(1):8. doi: 10.3390/md21010008 (PMC9866420; doi:10.3390/md21010008)
Supplement: Supplementary file 1 [file marinedrugs-21-00008-s001.zip › Table S4.pdf]

Table S4. *Vibrio alginolyticus* count on mussel samples of the Conero Riviera during 2021.

CONERO RIVIERA 2021					
		N°	*V.alginolyticus*		
**Sampling site**	**Sampling time**	**UFC g^{-1}**	**gyrB**	**NRPS**	**PKS**
Ancona nord	10/05/2021	$2.0x10^1$	+	*	*
Ancona Sud		$1.0x10^1$	+	*	*
Sirolo nord		$1.0x10^1$	+	*	*
Sirolo sud		$4.4x10^2$	+	*	*
Ancona nord	27/05/2021	$2.9x10^3$	+	+	-
Ancona Sud		$7.1x10^3$	+	+	-
Sirolo nord		$3.7x10^3$	+	-	-
Sirolo sud		$1.8x10^3$	+	-	-
Molo Portonovo (MP)	04/06/2021	0.0	*	*	*
Ancona nord	09/06/2021	$2.4x10^4$	+	+	-
Ancona Sud		$2.9x10^4$	+	+	-
Sirolo nord		$6.2x10^4$	+	-	-
Sirolo sud		$6.8x10^4$	+	+	-
Molo Portonovo (MP)	11/06/2021	0.0	*	*	*
Molo Portonovo (MP)	17/06/2021	$1.5x10^3$	+	+	-
Molo Portonovo (MP)	25/06/2021	$2.0x10^3$	+	+	-
Molo Portonovo (MP)	30/06/2021	$6.7x10^4$	+	-	-
Ancona nord	07/07/2021	$5.3x10^4$	+	-	-
Ancona Sud		$1.4x10^4$	+	-	+
Sirolo nord		$8.0x10^3$	+	-	+
Sirolo sud		$1.4x10^3$	+	-	-
Molo Portonovo (MP)	07/07/2021	$8.4x10^4$	+	-	-
Molo Portonovo (MP)	16/07/2021	$2.3x10^4$	+	+	-
Ancona nord	22/07/2021	$3.0x10^4$	+	-	-
Ancona Sud		$2.1x10^4$	+	-	-
Sirolo nord		$1.2x10^4$	+	-	-
Sirolo sud		$1.4x10^4$	+	-	-
Molo Portonovo (MP)	21/07/2021	$1.5x10^3$	+	-	-
Molo Portonovo (MP)	29/07/2021	$1.5x10^4$	+	+	-
Ancona nord	03/08/2021	$1.2x10^4$	+	+	-
Ancona Sud		$1.2x10^4$	+	+	-
Sirolo nord		$3.2x10^4$	+	+	-
Sirolo sud		$1.4x10^3$	+	+	-
Molo Portonovo (MP)	11/08/2021	$2.2x10^4$	+	*	*

* not performed
\+ detected
\- not detected
