# Supplementary material for: A Hotspot of TTX Contamination in the Adriatic Sea: Study on the Origin and Causative Factors
Source: Mar Drugs. 2022 Dec 22;21(1):8. doi: 10.3390/md21010008 (PMC9866420; doi:10.3390/md21010008)
Supplement: Supplementary file 1 [file marinedrugs-21-00008-s001.zip › Table S5.pdf]

Table S5. *Vibrio alginolyticus* count on various matrices from "Molo Portonovo" sampling point during 2021.

	MOLO PORTONOVO 2021		
Sampling date	sediment UFC g^{-1}	water UFC mL^{-1}	plankton-net UFC mL^{-1}
03/06/2021	$1.01x10^2$	$1.0x10^2$	$9.0x10^2$
11/06/2021	$2.5x10^2$	$2.0x10^1$	$4.5x10^2$
17/06/2021	$1.1x10^3$	$0.0x10^1$	$2.5x10^2$
24/06/2021	$9.0x10^2$	$5.0x10^1$	$1.8x10^3$
01/07/2021	$5.3x10^3$	$2.0x10^1$	$5.7x10^2$
08/07/2021	$1.3x10^3$	$2.3x10^2$	$4.6x10^4$
15/07/2021	$6.0x10^4$	$7.4x10^1$	$1.3x10^3$
23/07/2021	$1.1x10^4$	$2.4x10^2$	$4.0x10^2$
31/07/2021	$3.4x10^3$	$1.3x10^2$	$8.5x10^2$
06/08/2021	$6.7x10^3$	$5.3x10^1$	$4.2x10^2$
