# Supplementary material for: A Hotspot of TTX Contamination in the Adriatic Sea: Study on the Origin and Causative Factors
Source: Mar Drugs. 2022 Dec 22;21(1):8. doi: 10.3390/md21010008 (PMC9866420; doi:10.3390/md21010008)
Supplement: Supplementary file 1 [file marinedrugs-21-00008-s001.zip › Table S6.pdf]

Table S6. Pearson's correlation between dinoflagellate taxa abundances and TTX concentration in mussels, water temperature (T), salinity (S), DIN, $Si(OH)_4$ and PO_4^{3-} values. Values indicated in red are significant at $p < 0.05$, those in italic are significant at $p < 0.01$, those in bold italic are significant at $p < 0.001$. * indicates obligate heterotrophic dinoflagellates.

**Taxa**	**TTX**	**T**	**S**	**DIN**	**Si(OH)$_4$**	**PO$_4^{3-}$**
Akashiwo sanguinea (K.Hirasaka) Gert Hansen & Moestrup	-0.15	0.39	0.31	-0.19	0.04	-0.29
Tripos extensum (Gourret) F.Gómez	***0.98***	-0.38	-0.12	0.64	0.16	***0.92***
Tripos furca (Ehr.) F.Gómez.	0.04	0.13	-0.16	-0.13	0.00	0.04
Tripos fusus (Ehr.) F.Gómez	0.42	-0.01	-0.50	0.11	-0.23	0.52
Tripos muelleri Bory.	-0.06	0.22	0.08	0.39	0.49	0.01
Dinophysis caudata Saville-Kent	-0.19	0.34	-0.07	-0.25	-0.29	0.17
Dinophysis sacculus Stein	-0.09	-0.32	0.13	-0.35	0.08	-0.20
Gonyaulax polygramma Stein	0.07	0.10	-0.02	0.03	0.12	0.13
Gonyaulax spinifera (Clap. & Lach.) Diesing	0.63	-0.44	-0.71	0.13	-0.39	0.51
Mesoporos perforatus (Gran) Lillick	-0.16	0.22	0.32	-0.18	0.06	-0.17
**Noctiluca scintillans* (Macartney) Kofoid & Swezy	***0.95***	-0.31	-0.12	0.61	0.20	***0.92***
Oxytoxum crassum J.Schiller	***0.98***	-0.38	-0.12	0.64	0.16	***0.92***
Oxytoxum laticeps J.Schiller	0.07	0.10	-0.02	0.03	0.12	0.13
Oxytoxum scolopax Stein	0.07	0.10	-0.02	0.03	0.12	0.13
Phalacroma rotundatum (Clap. & Lach.) Kof. & Michener	-0.08	-0.05	***-0.90***	-0.29	-0.72	-0.13
Podolampas spinifera Okamura	-0.19	0.34	-0.07	-0.25	-0.29	0.17
Prorocentrum micans Ehr.	0.42	-0.26	***-0.89***	0.05	-0.58	0.33
Prorocentrum cordatum (Ostenfeld) J.D.Dodge,	-0.10	0.30	0.33	-0.27	0.15	-0.26
Prorocentrum triestinum Schiller	0.07	0.02	0.08	-0.12	0.20	0.06
**Protoperidinium bipes* (Paul.) Bal.	-0.04	-0.19	0.33	0.18	0.45	0.04
**Protoperidinium* cf. *steinii* (Jorg.) Bal.	***0.88***	-0.33	-0.52	0.46	-0.13	0.83
**Protoperidinium divergens* (Ehr.) Bal.	-0.36	0.45	-0.24	-0.18	-0.25	-0.14
**Protoperidinium pellucidum* Bergh	***0.98***	-0.38	-0.12	0.64	0.16	***0.92***
**Protoperidinium quinquecorne* (Abé) Balech	-0.10	0.05	***-0.92***	-0.33	-0.73	-0.06
**Protoperidinium* sp.	***0.89***	-0.24	-0.13	0.56	0.17	***0.93***
