# Supplementary material for: A Hotspot of TTX Contamination in the Adriatic Sea: Study on the Origin and Causative Factors
Source: Mar Drugs. 2022 Dec 22;21(1):8. doi: 10.3390/md21010008 (PMC9866420; doi:10.3390/md21010008)
Supplement: Supplementary file 1 [file marinedrugs-21-00008-s001.zip › Table S7.pdf]

Table S7 HILIC-MS/MS method for TTXs analysis: LC, MS parameters and transitions in Multiple Reaction Monitoring (MRM).

**LC PARAMETERS**			
**Column**	Glycan BEH Amide 130 Å 1.7μm, 2.1x150 mm (Waters)		
**Injection Volume**	2 μL		
**T° Column manager**	60 °C		
**T° Sample manager**	6 °C		
**Mobile phase A**	500 mL H_2O + 300 μL NH_4OH + 75 μL CH_2O_2		
**Mobile phase B**	700 mL CH_3CN + 300 mL H_2O + 100 μL CH_2O_2		
**Time(min)**	**Flow (mL/ min)**	**A (%)**	**B (%)**
0.00	0.4	2	98
7.00	0.4	2	98
9.50	0.4	50	50
11.00	0.5	50	50
11.50	0.5	2	98
12.00	0.6	2	98
12.50	0.6	2	98
13.00	0.4	2	98
14.00	0.4	2	98

**MS/MS PARAMETERS**	
**Source type**	EI
**Capillary**	3,5 kV
**Desolvatation Temp.**	600 °C
**Desolvatation**	1000 L/Hr
**Cone**	150 L/Hr
**Source Temp.**	150 °C
**Ionization mode**	ES+

MRM TRANSITIONS

**Compound**	**Prec. ion (m/z)**	**Prod. ion (m/z)**	**Dwell (sec)**	**Cone (V)**	**CE (V)**
**TTX/4-epi TTX**	320.1	302.1	0.026	40	30
		162.1	0.026	40	40
**11-nor TTX-6-ol/ 6,11-dideoxyTTX**	290.1	272.1	0.026	40	30
		162.1	0.026	40	30
**5-DeoxyTTX/ 11-Deoxy TTX**	304.1	286.1	0.026	40	30
		162.1	0.026	40	30
**11-oxo-TTX**	336.1	318.1	0.026	40	30
		300.1	0.026	40	30
**5,6,11 TrideoxyTTX**	272.1	254.1	0.026	40	30
		162.1	0.026	40	30
**4,9-anhydroTTX**	302.1	256.1	0.026	40	30
		162.1	0.026	40	30
