# Supplementary material for: A Hotspot of TTX Contamination in the Adriatic Sea: Study on the Origin and Causative Factors
Source: Mar Drugs. 2022 Dec 22;21(1):8. doi: 10.3390/md21010008 (PMC9866420; doi:10.3390/md21010008)
Supplement: Supplementary file 1 [file marinedrugs-21-00008-s001.zip › Table S8.pdf]

Table S8 Reagents, reaction mix and protocols for PCR on *Vibrio* spp.

PCR ANALYSIS

Fragments	***Primers***		***Target***
gyrB (560bp)	AlgF1	5′-TCA GAG AAA GTT GAG CTA ACG ATT-3′	*V. alginolyticus*
	AlgR1	5′-CAT CGT CGC CTG AAG TCG CTG T -3′	
NRPS (300bp)	A2gamF	5′-AAG GCN GGC GSB GCS TAY STG CC-3′	*V. alginolyticus*
	A3gamR	5′-TTG GGB IKB CCG GTS GIN CCS GAG GTG - 3′	
PKS (300bp)	DKF	5′-GTG CCG GTN CCR TGN GYY TC-3′	*V. alginolyticus*
	DKR	5′-GCG ATG GAY CCN CAR CAR MG -3′	

Amplification Protocols				***Reaction Mix***	
Denaturation	94 °C x 4 min	94 °C x 3min	94 °C x 2 min	Ultrapure water	12.45µL
Denaturation	94 °C x 30 s	94 °C x 1 min	94 °C x 1 min	Buffer Go Taq DNA Flexi polimerase 5x (promega)	5.0 µL
Annealing	58 °C x 30 s **32 cycles**	60 °C x 1 min **35 cycles**	60 °C x 1 min **30cycles**	$MgCl_2$ (25mM)	2.0 µL
Extension	72 °C x 1 min	72 °C x 2 min	72 °C x 1 min	$dNTP_s$ (2,5 mM)	2.0 µL
Extension	72 °C x 8 min	72 °C x 7 min	72 °C x1 min	Primer (20µm)	0.5 µL
Target	***gyrB***	**NRPS**	**PKS**	Primer (20µm)	0.5 µL
	[54]	*[55]*	*[56]*	GO Taq Flexi DNA polymerase (5u/µl) (promega)	0.05 µL
Positive CTRL+	*V. alginolyticus* ATCC 33787	*V. parahaemolyticus* ATCC 17802		Volume mix	22.5 µL
				DNA	2.5 µL
Negative CTRL-	Ultrapure water	Ultrapure water		Final volume	25.0 µL
